# Supplementary material for: Exportin Crm1 is repurposed as a docking protein to generate microtubule organizing centers at the nuclear pore
Source: eLife. 2018 May 29;7:e33465. doi: 10.7554/eLife.33465 (PMC6008054; doi:10.7554/eLife.33465)
Supplement: Transparent reporting form [file elife-33465-transrepform.docx]

***eLife’s* transparent reporting form**

We encourage authors to provide detailed information *within their submission* to facilitate the interpretation and replication of experiments. Authors can upload supporting documentation to indicate the use of appropriate reporting guidelines for health-related research (see [EQUATOR Network](http://www.equator-network.org/%20)), life science research (see the [BioSharing Information Resource](https://biosharing.org/)), or the [ARRIVE guidelines](http://www.plosbiology.org/article/info:doi/10.1371/journal.pbio.1000412) for reporting work involving animal research. Where applicable, authors should refer to any relevant reporting standards documents in this form.

If you have any questions, please consult our Journal Policies and/or contact us: [editorial@elifesciences.org](mailto:editorial@elifesciences.org).

**Sample-size estimation**

- You should state whether an appropriate sample size was computed when the study was being designed
- You should state the statistical method of sample size computation and any required assumptions
- If no explicit power analysis was used, you should describe how you decided what sample (replicate) size (number) to use

Please outline where this information can be found within the submission (e.g., sections or figure legends), or explain why this information doesn’t apply to your submission:

Sample size estimation and power analysis were not required for this work. Experiments involved yeast cells with defined genotypes. In instances where statistical analysis is used, it is easy to obtain and score large numbers of cells.

**Replicates**

- You should report how often each experiment was performed
- You should include a definition of biological versus technical replication
- The data obtained should be provided and sufficient information should be provided to indicate the number of independent biological and/or technical replicates
- If you encountered any outliers, you should describe how these were handled
- Criteria for exclusion/inclusion of data should be clearly stated
- High-throughput sequence data should be uploaded before submission, with a private link for reviewers provided (these are available from both GEO and ArrayExpress)

Please outline where this information can be found within the submission (e.g., sections or figure legends), or explain why this information doesn’t apply to your submission:

Numbers of replicate experiments are described in Materials and Methods. A definition of biological replicates is presented in Materials and Methods. Technical replicates are not applicable. Outliers are not applicable, as all data were included in analyses.

**Statistical reporting**

- Statistical analysis methods should be described and justified
- Raw data should be presented in figures whenever informative to do so (typically when N per group is less than 10)
- For each experiment, you should identify the statistical tests used, exact values of N, definitions of center, methods of multiple test correction, and dispersion and precision measures (e.g., mean, median, SD, SEM, confidence intervals; and, for the major substantive results, a measure of effect size (e.g., Pearson's r, Cohen's d)
- Report exact p-values wherever possible alongside the summary statistics and 95% confidence intervals. These should be reported for all key questions and not only when the p-value is less than 0.05.

Please outline where this information can be found within the submission (e.g., sections or figure legends), or explain why this information doesn’t apply to your submission:

When used, statistical analysis methods/tests are described in figure legends. Raw data values are shown. Values for N and p-values are given in figures or figure legends. In fluorescence images of localization of GFP-tagged Mto1 variants/fragments, Nup146-3mCherry, Alp7-3GFP and GST-NLS-mCherry, each image shows only a few cells. Therefore, to indicate the consistency of protein localization in each strain, images are accompanied by values describing the percentage of cells displaying the particular localization concerned, as well as the number of cells counted (N). In nearly all cases, over 100 cells were examined, and in nearly all cases, percentages were close to “all or nothing” (i.e. close to zero, or greater than 90%). As these values are presented only to give an overall indication of localization, they were not subjected to statistical analysis.

(For large datasets, or papers with a very large number of statistical tests, you may upload a single table file with tests, Ns, etc., with reference to sections in the manuscript.)

**Group allocation**

- Indicate how samples were allocated into experimental groups (in the case of clinical studies, please specify allocation to treatment method); if randomization was used, please also state if restricted randomization was applied
- Indicate if masking was used during group allocation, data collection and/or data analysis

Please outline where this information can be found within the submission (e.g., sections or figure legends), or explain why this information doesn’t apply to your submission:

Samples in this study consist of yeast strains expressing different protein variants and/or containing specific mutations, in otherwise isogenic backgrounds. Samples were not allocated into specific experimental groups.

**Additional data files (“source data”)**

- We encourage you to upload relevant additional data files, such as numerical data that are represented as a graph in a figure, or as a summary table
- Where provided, these should be in the most useful format, and they can be uploaded as “Source data” files linked to a main figure or table
- Include model definition files including the full list of parameters used
- Include code used for data analysis (e.g., R, MatLab)
- Avoid stating that data files are “available upon request”

Please indicate the figures or tables for which source data files have been provided:

For each figure relating to mass spectrometry, mass spectrometry datasets and dataset summaries from all replicate experiments associated with the figure are presented in a file containing Excel spreadsheets. For Figs. 1F, 3C, 4D, and 6F (and associated supplementary figures), these correspond to Supplementary Files 3-6, respectively. Mass spectrometry data from a preliminary SILAC experiment (not shown in any figure) are presented in an Excel spreadsheet in Supplementary File 2.
